# The Effects of Coaching Patients to List Questions Before Visiting Cancer Specialists: Retrospective Evaluation of Visit Preparation in a Rural, Underserved Setting

**DOI:** 10.2196/jopm.8949

**Published:** 2017-08-22

**Authors:** Jeffrey K Belkora, Marijoyce Naguit, Lauren Stupar, James Wiley, Shelley Volz, Sara O'Donnell

**Keywords:** Visit preparation, self-efficacy, anxiety, question list, patient support, community-based participatory research, psycho-oncology.

## Abstract

**Background:**

A community-based organization implemented an evidence-based intervention to help rural cancer patients list questions before oncology visits.

**Objective:**

Was the question-listing intervention effective in reducing anxiety and increasing decision self-efficacy?

**Methods:**

The organization surveyed patients on decision self-efficacy (273 respondents, 99% response rate) and anxiety (190, 68%) before and after question-listing interventions delivered from 2006 – 2011. We analyzed responses using two-sided paired t-tests at 5% significance and conducted linear regression to identify significant predictors of change. We examined predictors related to the patient (location, demographics, disease status and baseline decision self-efficacy and anxiety); the intervention (including interventionist case volume); and the visit (including type of doctor seen).

**Results:**

Question-listing was associated with higher mean decision self-efficacy (2.70/3.43 pre/post, 1-4 min-max, *P*<.001) and lower mean anxiety (7.26/5.87, 1-10 min-max, *P*<.001). Significant predictors of change in decision self-efficacy included: patient location; interventionist case volume; baseline decision self-efficacy and anxiety. Higher baseline anxiety was also associated with reductions in anxiety.

**Conclusions:**

In a sustained community-based implementation, the intervention helped patients prepare for oncology visits. Patients reported higher self-efficacy and lower anxiety.

 

## Introduction

People facing cancer are known to experience communication barriers that impede their ability to address their information needs with their most trusted sources of information, namely their physicians [1,2,3].  Researchers have proposed various approaches to helping patients prepare for medical visits in order to overcome these barriers and obtain personalized information. These interventions were summarized in a recent systematic review [4]. For example, Cegala and colleagues developed a comprehensive model (PACE) that teaches the patient to provide information, ask questions, check or clarify understanding, and express concerns. A common theme across such visit preparation interventions is the importance of helping patients ask questions. Roter, Butow, Cegala, and colleagues have documented that having a written list of questions is associated with an increase in the number and range of questions that patients ask [1,4,5,6,7], with minimal or no harm [1,8,9,10].

Based on this evidence, one of the authors (JB) developed a process for coaching patients to identify and write down questions for upcoming visits with specialists [11]. An evaluation found the question-listing intervention efficacious [12], and researchers implemented it at the University of California, San Francisco (UCSF) breast care center [13,14], where it has been sustained by internal and external funds since 1998 [15,16]. The intervention has spread to other urban or academic settings, where it has been associated with an increase in decision self-efficacy [13,17,18,19], i.e. patient confidence about making decisions with providers [20].

In 1998, a rural patient support organization in Northern California adopted question-listing based on a recommendation from a nurse who moved from urban Palo Alto to rural Mendocino, CA. This community-based organization, known as the Cancer Resource Centers of Mendocino County (hereafter, the resource center), attended annual training at UCSF starting in 2000. [21] In 2003 the resource center embarked on a community-based participatory research program with UCSF to adapt, expand, and evaluate its question-listing service. In our initial evaluation, we found high levels of satisfaction among the existing clientele of the resource center, [22] and successfully adapted the intervention to the needs of the diverse, rural population [23], including delivery by telephone [17].

Since 2006, the resource center routinely collects responses from all patients to decision self-efficacy and anxiety surveys before and after the question-listing intervention. Therefore, in 2012 the Executive Director (author SO) suggested analyzing all these surveys to learn about effects on anxiety as well as decision self-efficacy.

The authors believed this presented a good opportunity to evaluate the effects on psychosocial outcomes of an intervention implemented in a rural, medically underserved community setting. University researchers (authors JB, MN, LS, JW, and SV) joined forces with the resource center (author SO) to review program records over a five-year period (2006-2011). We asked the following questions:

1. Was the question-listing intervention associated with changes in decisional self-efficacy and anxiety?
2. Did changes in decision self-efficacy and anxiety vary across subsets, including patients who did not have breast cancer?
3. Were there any significant predictors of variation in decision self-efficacy and anxiety?

## Methods

### Decision Self-Efficacy (DSE)

Notice that the first subheading immediately follows the last heading. Subheadings under subheadings are also possible (see Statistical Analysis).

### Anxiety

The resource center measured anxiety using a single item, administered at the same time as the decision self-efficacy scale. The item measures anxiety on a scale of 1-10 by asking respondents to complete the statement “On a scale of 1-10 (1 being the lowest, 10 being the highest), my anxiety level is…” The resource center used a single item to minimize patient burden as was done in two prior studies [24,25] where a single item was found to be an acceptable substitute for a longer standardized scale. The rationale for measuring anxiety was the evidence that reducing the immediate anxiety and distress surrounding a cancer diagnosis can positively influence the patient’s trajectory through treatment and survivorship, including pain and fatigue, quality of life (bodily pain, physical function), treatment adherence, future cancer surveillance, health behaviors (e.g., exercise), self-care (e.g., managing lymphedema), immune function, and recurrence and survival [26-32]. As with decision self-efficacy, resource center leaders felt that their organization’s delivery of question-listing could directly influence anxiety as the first link in a longer causal chain, most of which was outside of their direct control.

### Predictor variables

**Figure 2:**
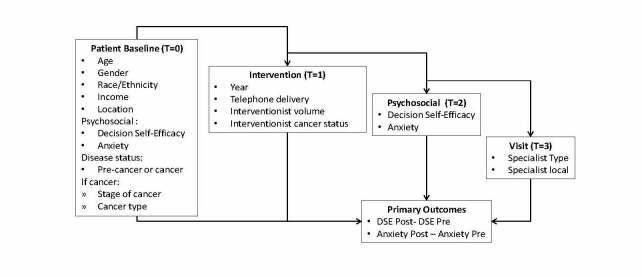
Predictor variables for decision self-efficacy (DSE) and anxiety.

In addition to decision self-efficacy and anxiety, we abstracted from resource center records information about respondent demographics:  Age (continuous), gender (male/female), ethnicity (white non-hispanic versus non-white), income (<= 250% of the federal income level versus >250%), location of first intervention (coastal versus inland office of the resource center); disease status (pre-cancer versus invasive cancer); and if diagnosed with invasive cancer, what stage (I-IV). We initially coded type of cancer into breast versus other, since our prior studies had focused exclusively on breast and we were curious about any differences versus all other cancers. We subsequently categorized type of cancer into 11 categories: breast, colorectal, digestive system, head and neck, hematologic, lung, ovarian, prostate, skin, urogenital, and other cancers. We captured service delivery characteristics including year of patients’ first access to intervention (2006-2011) and whether the intervention was delivered over the telephone (yes or no). There were 12 resource center employees who administered the question-listing intervention in the study period. Interventionist characteristics included intervention volume since 2003 (a continuous variable reflecting the employee’s experience administering the intervention) and whether the interventionist was a cancer survivor (yes or no). The interventionist characteristics were logically associated with each other and with location. The resource center was founded by cancer survivors, and so the longest-tenured and highest-volume interventionists were cancer survivors, and they worked in the inland office. Conversely, newer and lower-volume interventionists were not cancer survivors, and worked in the coastal office. We kept this multi-collinearity in mind when conducting our exploratory multivariable analyses. Finally, we gathered from program records information on the specialist type visited (medical oncologist, radiation oncologist, surgeon, other) and whether the specialist was local (local or non-local). See Figure 2.

 

 

 

### Intervention

with each other and with location. The resource center was founded by cancer survivors, and so the longest-tenured and highest-volume interventionists were cancer survivors, and they worked

### Question-Listing

Resource center employees routinely offer a question-listing intervention to clients with a cancer diagnosis who self-refer or are referred by health care professionals for community-based psychosocial support. The intervention consists of a structured interview in which the interventionist, a lay health worker, prompts a patient to articulate questions and concerns in preparation for a treatment discussion with a cancer specialist. The interventionist prepares a word-processed document paraphrasing and summarizing the patient’s questions and concerns. The patient takes away the printed question list to serve as a visual aid and agenda during the meeting with the doctor. The prompts and an example question list are available online [33,34] and in the literature [13], where the intervention is referred to as Consultation Planning.

 

### Interventionists

The resource center has two offices, one in Mendocino Village, on the coast, and the other in the town of Ukiah, inland. They are located an hour and half apart by car, and each location has its own staff. Assignment of the staff member who administered the question-listing service to clients was based on availability and proximity.

### Data Collection Procedures

**Figure 1:**
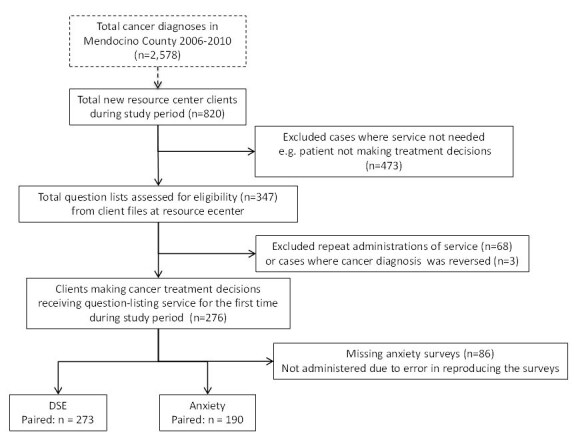
Flow chart for creation of analytic sample.

Following ethics approval from the UCSF committee on Human Research, author MN visited each resource center site and reviewed the paper files of every client that received question-listing. From these 347 files, author MN recorded demographic information obtained by the resource center upon patient registration at intake, and responses to the decision self-efficacy and anxiety questionnaires stored in the program records. This left us with 276 client files, 273 of whom had completed both pre and post surveys for decision self-efficacy (response rate = 99%), and 190 of whom had completed pre and post surveys for anxiety (response rate = 68%). See Figure 1. The surveys were collected immediately before and after the intervention by the interventionists.

** **

### Analysis Plan

#### Question 1: Was the Question-Listing Intervention Associated With Changes in Decisional Self-Efficacy and Anxiety?

We compared dotplots to examine whether the distributions of decision self-efficacy and anxiety had shifted; and scatterplots to examine the changes on a paired (pre/post) basis. We used the binomial sign test to test the null hypothesis of responses being as likely to go up or down, at a significance level of 5%. We used two-sided paired t-tests to compare, at a significance level of 5%, the overall pre and post mean decision self-efficacy and anxiety scores. We used a published algorithm to calculate Cohen’s d for paired data [35], adjusting for the correlation between pre and post scores.

#### Question 2: Did Changes in Decision Self-Efficacy and Anxiety Vary Across Subsets, Including Patients Who Did not Have Breast Cancer?

For binary and other categorical variables, we tabulated and compared the mean change scores within each subset level, using a paired t-test of the null hypothesis of no change at a significance level of 0.05. This helped us understand variation across subsets. To further assess predictors of variation, we conducted simple linear regression to assess whether each categorical or continuous predictor was significantly associated in linear fashion with either decision self-efficacy or anxiety change scores, testing whether the coefficient was significantly different from zero at a significance level of 0.05. Here and elsewhere, we did not correct for multiple significance tests, as we considered these analyses descriptive and exploratory.

#### Question 3: Were There Any Significant Predictors of Variation in Decision Self-Efficacy and Anxiety?

From our simple linear regression results, we selected the predictors with a p-value less than 0.05. We entered all these into a multivariable linear regression model, then iteratively removed the least significant predictor, until we had a parsimonious model.  We interpreted our multivariable regression results as exploratory and used them to refine hypotheses and measurement strategies for future studies.

## Results

### Sample characteristics

Our survey respondents were majority female (76%), in part because 50% of the clients overall had breast cancer. Most (89%) were white, non-Hispanic. Many (39%) were low-income (under 2.5 times the federal poverty level). Clients ranged from 26 to 91 in age, with a mean and median of 60. Eighty-six clients did not answer the anxiety question. This was due to an error in reproducing the paper surveys. The non-respondents to anxiety resembled respondents in terms of key demographics (83% female, 92% white, 34% low-income).

#### Question 1: Was the Question-Listing Intervention Associated With Changes in Decisional Self-Efficacy and Anxiety?

**Figure 3:**
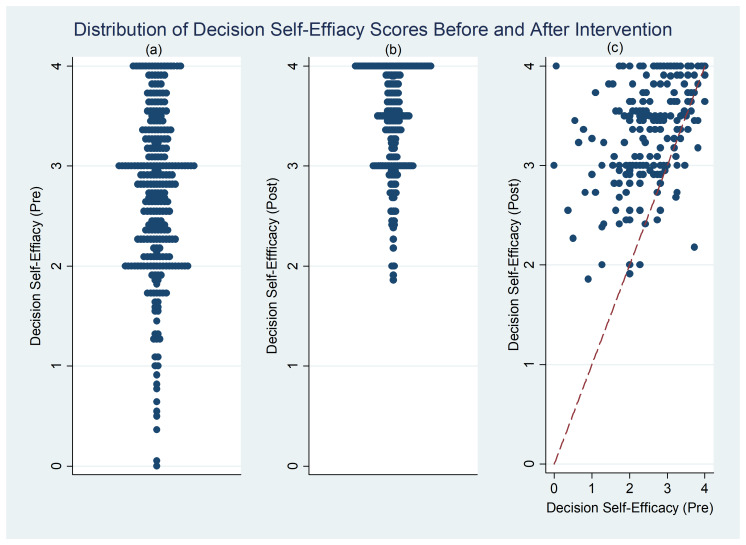
Distribution of decision self-efficacy scores before and after intervention.

Dotplot graphs of the decision self-efficacy pre (Figure 3 a) and post (Figure 3 b) show an upward shift in the distribution, reflecting improvement. A scatterplot (Figure 3 c) reveals that on a paired basis, most decision self-efficacy scores went up (221 out of 273, or 81%) while 32/273 stayed the same (12%) and 20 out of 273 scores (7%) went down. This is significantly different from the null hypothesis of an equal number of scores going up or down (sign test *P*<.001). The decision self-efficacy scale performed well in terms of psychometrics in this sample:  we calculated a value of 0.96 for Cronbach’s alpha for the pre-intervention responses to Decision Self-Efficacy. Cronbach’s alpha was 0.93 for the post-intervention responses [36].

**Figure 4:**
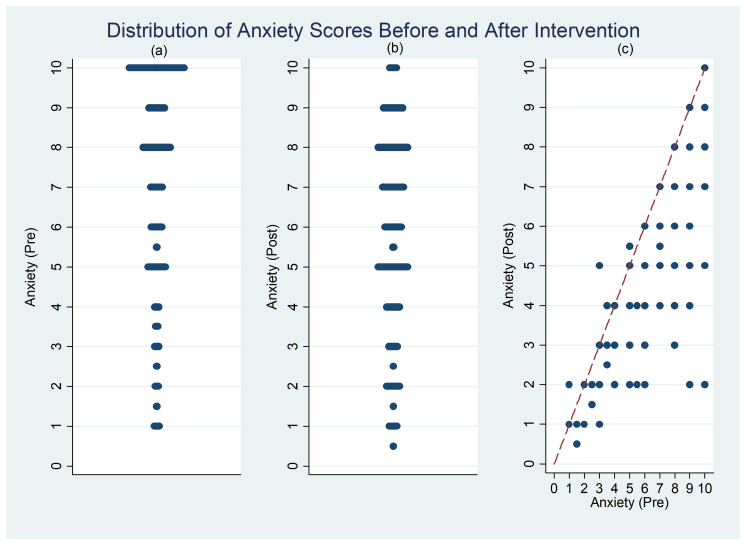
Distribution of anxiety scores before and after intervention.

Dotplot graphs of anxiety responses pre (Figure 4 a) and post (Figure 4 b) show a downward shift in the distribution, reflecting improvement. A scatterplot (Figure 4 c) reveals that 136 out of 190 (72%) anxiety scores went down, 49 (26%) stayed the same, and only 5 (2%) went up. This is significantly different from the null hypothesis of an equal number of scores going up or down (sign test *P*<.001).

The overall mean decision self-efficacy score rose from 2.70 pre to 3.43 post, an increase of 0.73, which translated to an effect size (Cohen’s d) of 1.04 [35]. A two-sided paired t-test with alpha 0.05 shows this increase is significant (*P*<.001) and the 95% confidence interval for the change was 0.65 to 0.82. The mean anxiety dropped from 7.27 pre to 5.87 post,a decrease of 1.40, which translated to an effect size (Cohen’s d) of 1.00. A two-sided paired t-test with alpha 0.05 shows this decrease was significant (*P*<.001) and the 95% confidence interval for the change was -1.60 to -1.20. The question-listing intervention was associated with a consistent and large effect on the patients.

 

#### Question 2: Did Changes in Decision Self-Efficacy and Anxiety Vary Across Subsets, Including Patients Who Did not Have Breast Cancer?

**Figure 5:**
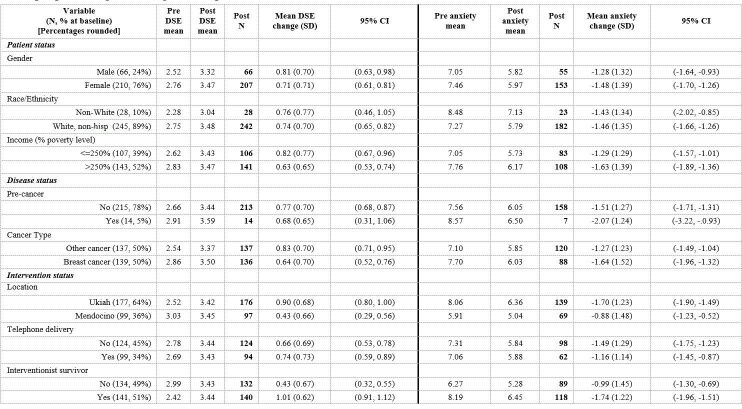
Decision self-efficacy (DSE) and anxiety for subsets defined by dichotomous predictor variables.

Figure 5 shows the results for subsets defined by dichotomous predictor variables. Discussed below are the multi-category variables (stage and type of cancer, interventionist, and type of specialist consulted) and the continuous variables (age, year and interventionist volume). We found that mean decision self-efficacy increased and anxiety decreased significantly from pre to post in all of the subsets defined by our dichotomous variables.

For the subsets defined by multi-category variables, regarding the outcome of change in decision self-efficacy, we found that interventionist and cancer stage were significant predictors, while type of cancer and type of specialist were not. For anxiety, among the multi-category variables, only interventionist was a significant predictor, meaning that the amount of anxiety reduction varied significantly according to which staff member delivered the question-listing intervention.

Using simple linear regression, we found that nine dichotomous predictors were significant predictors of change in decision self-efficacy. Among these, five had negative coefficients, meaning that an increase in the predictor would be associated with a decreased change in decision self-efficacy. Specifically, being more than 250% above the poverty level (versus below), having breast cancer (versus other cancers), higher baseline decision self-efficacy, living in the Mendocino coastal region (versus inland Ukiah), and seeing a local (versus non-local) specialist were all associated with decreased change in decision self-efficacy (less improvement).

Conversely, higher baseline anxiety, receiving the intervention by telephone, or from an interventionist who was a cancer survivor or had a higher volume of experience, all were associated with larger gains in decision self-efficacy, and therefore predictive of greater improvement.

**Figure 6:**
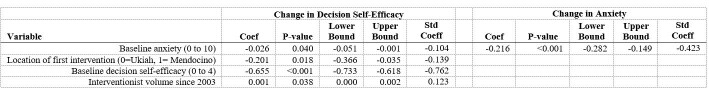
Summary of significant predictor variable performance in multivariable linear regression.

We found that six dichotomous predictors were significantly associated with the change scores for anxiety. Among these, three were negatively correlated, meaning that an increase in the predictor was associated with greater improvement (reductions) in anxiety. Specifically, higher anxiety, and receiving the intervention from an interventionist who was either a cancer survivor or had a higher volume of experience were all associated with greater improvements in anxiety. Conversely, increasing baseline decision self-efficacy, living in coastal Mendocino (versus Ukiah), and receiving the intervention by telephone were all associated with smaller improvements.  See Figure 5 and Figure 6 (tabs 1 and 2)

 

 

 

#### Question 3: Were There Any Significant Predictors of Variation in Decision Self-Efficacy and Anxiety?

Compared to our simple linear regression, considering predictor variables together simultaneously meant that we dropped five variables that previously were significant predictors of change in decision self-efficacy: income; cancer stage; cancer type; and whether the interventionist was a cancer survivor. The final model therefore included baseline anxiety (a continuous variable on a scale of 0-10 with responses ranging from 1-10) and baseline decision self-efficacy (scale and range of 0 to 4); location (coastal versus inland offices of the resource center) and interventionist volume .  Among the 12 interventionists, volume of experience administering question-listing sessions ranged from 1 to 89.

Location was correlated with interventionist volume (correlation coefficient r=-0.62) and also with whether the interventionist was a cancer survivor (r=-0.77). We therefore considered alternative multivariable models, including one substituting location for cancer survivor, but our final model explained the largest proportion of the variance (65%) so we adopted it as the best fit.

Considering predictor variables together simultaneously also led us to drop five variables that previously were significant predictors of change in anxiety: baseline decision self-efficacy; location; telephone delivery; interventionist cancer survivor; and volume. The final model included only baseline anxiety. See Figure 6 for details.

## Discussion and Conclusions

For each of the study questions, we now interpret the findings, with special attention to surprising or otherwise interesting results, and in comparison with prior publications in the literature.

### Question 1: Was the Question-Listing Intervention Associated With Changes in Decisional Self-Efficacy and Anxiety?

Each outcome saw a marked shift, reflecting improvement, in the distribution from baseline to post-intervention. These results are consistent with other studies that have found improvements in decision self-efficacy. The same intervention was associated with improved decision self-efficacy in this community setting among breast cancer patients,[17] among breast cancer patients in a US academic setting,[13] among blood cancer patients in a US community setting,[18] and among prostate cancer patients in Scotland.[19] Related interventions, such as decision aids for hormone replacement therapy,[37,38] colorectal cancer[39] and prostate cancer,[40] have also been associated with improvements in decision self-efficacy. Our results therefore extend to a rural population for the first-time results that have been found in urban settings.

We were interested to find reductions in anxiety, as there have been mixed results reported in the literature. In a study of our intervention with blood cancer patients in the US [18], we saw a reduction in the same measure of anxiety from a mean of 4.6 pre-intervention to 3.5 post-intervention, a similar relative reduction (24%) as we saw in this study (7.26 to 5.87 or 19%), but at lower absolute levels of anxiety. Anxiety remained higher after our intervention than it was before the intervention in the prior study. One possible explanation for this is that patients who received our question listing intervention were usually preparing for a visit that was occurring very soon after their diagnosis, having been referred in many cases to the resource center by the diagnosing physician. Therefore, while our intervention was associated with significant reductions in their anxiety, patients were likely still very anxious because they had not yet discussed treatment options and outcomes with a specialist. In the prior study, patients had self-referred to a resource center that typically does not see patients immediately upon their diagnosis. These patients may have had more time to adjust to their cancer diagnosis. Based on this finding, we plan to measure referral source and time since diagnosis in future studies of our intervention. Another possibility is that patients diagnosed with cancer in this rural, medically underserved community may experience higher levels of anxiety than patients in the more urban setting of the prior study, due to a disparity in their access tomedical care.

### Question 2: Did Changes in Decision Self-Efficacy and Anxiety Vary Across Subsets, Including Patients Who Did not Have Breast Cancer?

Given that almost all respondents reported paired improvements, it makes sense that all of the subsets with more than 7 patients reflected mean improvements in the outcomes. This finding suggests that the intervention is patient-centered enough to produce good effects across subsets of patients, including both genders, across ages and income levels, cancer types and stages, and various interventionists (including some who were cancer survivors and some who were not) delivering the intervention either in person or by telephone. There were relatively few non-white clients in the sample (28 or 10%). These clients had lower decision self-efficacy and higher anxiety at baseline than whites, but reported the same improvements as whites. This is consistent with our prior examination of Hispanic ethnicity patients reviewing decision aids in an academic medical setting [41]. There we found that Hispanic patients reported higher baseline decisional conflict than non-Hispanics, while reporting larger decreases, which represent improvements on that measure.

 

### Question 3: Were There Any Significant Predictors of Variation in Decision Self-Efficacy and Anxiety?

Patients starting in different states reported different intervention effects, making the baseline scores significant predictors of the change scores. One explanation may be that patients reporting lower baseline anxiety and higher baseline decision self-efficacy gave themselves less leeway to report improvement, without yet knowing how they would feel after the intervention. Conversely, patients with higher baseline anxiety and lower baseline decision self-efficacy left themselves more room to report improvement. As a result, patients with the same subjective response to the intervention could have reported different improvement levels, based on whether they were blocked from reporting the full perceived effect by a floor or ceiling when they reached the limit of the scale. Thus, the finding that the baseline scores were significant predictors of the change score could be spurious. Future researchers may want to consider adding retrospective pre/post assessments to disentangle the floor or ceiling effects from the perceived intervention effects.

Patient location referred to the fact that the resource center has two geographically distinct sites, one inland office in Ukiah, and one coastal office in Mendocino village. Residents on the coast reported higher baseline decision self-efficacy (mean 3.03 versus 2.52 for Ukiah) and lower baseline anxiety (mean 5.91 versus 8.06 for Ukiah). This played in to the floor and ceiling effects described above. In addition, location was highly correlated with interventionist volume and whether the interventionist was a cancer survivor. This collinearity means it was difficult to interpret our regression results, which remain exploratory and hypothesis-generating.

### Limitations and Strengths

The strengths of this study include that it examined the effectiveness of an evidence-based intervention translated into a rural, underserved community setting and sustained there. Our intervention adds to the literature because, in contrast with self-administered prompt sheets, it is administered by a trained facilitator who helps patients verbally brainstorm a personalized list of questions expressed in their own words.

Our study represents practice-based evidence with high external validity, meaning the study conditions were representative of real-world conditions in the way the intervention was delivered, and the range of clients. One of the study outcomes, decision self-efficacy, is part of a conceptual model, the Ottawa Decision Support Framework, that relies on self-efficacy as a known predictor of behavior and health outcomes. We reported on responses to a survey instrument that has documented psychometric properties and that has been used in other studies with similar populations.

Our second study outcome, anxiety, was measured using a study-specific, single-item survey instrument. Other studies that compared single item anxiety measures similar to ours to a 20-item standardized scale found that the single item was an acceptable substitute [24,25]. However, longer instruments are generally more reliable measures of psychological constructs such as anxiety. In addition, we had a large number (86 out of 276) of missing responses to the anxiety questions due to errors in reproducing the anxiety question on resource center evaluation forms. We cannot know whether the non-respondents would have reported different results than what we found from respondents.

Both of our outcome measures were near-term patient-reported outcomes. We do not have direct evidence from this study of longer lasting effects. Leaders of the community agency implementing the intervention felt that these were the most appropriate outcomes for their program to track because they could be closely linked, conceptually and chronologically, to the intervention. In addition, community representatives felt that improving decision self-efficacy and reducing anxiety are important components of patient-centered care, and that improving these outcomes would be worthwhile even if the intervention had no longer-term effects. This is consistent with the view of the Institute of Medicine that getting through treatments more psychologically supported is an important end in itself[42] because it improves the patient experience in health care. Nevertheless, future studies should examine downstream effects of question-listing, including whether it changes treatment decisions, adherence to those decisions, clinical outcomes, overall resource use, and quality of life.

Other weaknesses of this study include that it consisted of a pre/post design without a control group. The main threat to internal validity for this design is that the respondents might have reported similar improvements through the simple passage of time. This maturation bias may have been mitigated by the fact that program staff administered the survey instruments immediately before and immediately after the intervention. However, the fact that the same people administered the intervention and the surveys creates a potential motivational or social agreement bias, as respondents might feel socially beholden to the interventionists and respond with a desire to please them.

Our data set included responses to survey items that asked patients to rate, quantitatively, their decision self-efficacy and anxiety. Thus, we lacked qualitative data that might have added more insight to our findings.

Some of our analytic results may have been distorted by collinearity. For example, two long-time (and therefore high volume) program staff who are breast cancer survivors work at the inland (Ukiah) office, whereas more recent (and therefore lower volume) program staff who are not cancer survivors work in the coast office (Mendocino Village). Thus, the location is strongly associated with volume of experience and survivorship status, and all are associated with the change in decision self-efficacy. This kind of collinearity can distort the results of a linear regression, which is predicated on linearly independent variables.

We believe that these issues do not threaten the overall findings, which show robust intervention effects across subsets. However, they reinforce the fact that our regression results should be interpreted as exploratory and hypothesis generating. In addition, we do not know what particular features of the intervention, if any, may have contributed most to the effects on decision self-efficacy and anxiety.

 

### Current Translational Status and Future Directions

The question-listing intervention is now being implemented and sustained as part of routine care in several academic and community settings. It was first implemented at UCSF and has been sustained there by internal and external funds since 1998 as part of patient-centered care initiatives. The resource center featured in this study also has sustained the delivery of question-listing services by paid staff since 1998. The resource center provides all of its services free of charge through philanthropic grants and donations from foundations, corporations, and individuals. Since 2012, another non-profit agency, the Cancer Support Community, has also sustained with philanthropic support its implementation of our question-listing intervention. It delivers the intervention across the USA through a nationwide toll-free telephone line, in English and Spanish, free of charge to people with cancer, as well as in-person at physical locations in 33 communities [43]. Since 2013, the Center for Shared Decision Making at Dartmouth-Hitchcock Medical Center has also sustained its implementation of question-listing with philanthropic as well as internal budgetary support [44].

These organizations share a common motivation to implement a visit preparation intervention that addresses patient needs for short-term assistance with navigating treatment decision-making consultations. Helping patients ask questions is, in their view, an ethical imperative to advance the patient-centered outcomes of informed consent and informed choice. 

These agencies demonstrate the viability of implementing such a question-listing service in the voluntary sector with philanthropic support. This leaves open the question as to whether other payers, such as private or government health care plans, will fund this or similar question-listing or other visit preparation services. Such payers are increasingly looking for interventions that improve patient experience, improve outcomes, and increase health care economic value. As revealed in multiple studies cited above, question-listing does improve the patient experience of care. It remains to be seen whether question-listing contributes to different patient choices, resource use, or long-term outcomes. We also foresee the need to better understand what questions patients ask through content analysis, and what features of the intervention are most responsible for its effectiveness.

Our view is that question-listing merits wider adoption because it improves the patient experience of care. Our research agenda now turns to mechanisms for reducing the cost of delivering the intervention, to reduce barriers to adoption. We are exploring the feasibility of delivering our question-listing intervention on a large scale using trainees who will earn academic credit and gain practical experience while serving patients at low cost [15,45].

 

** **
